# Ultrasonography or direct radiography? A comparison of two techniques to detect dorsal screw penetration after volar plate fixation

**DOI:** 10.1186/s13018-018-0774-5

**Published:** 2018-04-03

**Authors:** Yunus Oc, Bekir Eray Kilinc, Anıl Gulcu, Ali Varol, Rodi Ertugrul, Adnan Kara

**Affiliations:** 1 Sisli Hamidiye Etfal Training and Research Hospital, Halaskargazi Cad., Etfal Sk, Şişli, 34371 Istanbul, Turkey; 2 Golhisar State Hospital, Fatih Mahallesi, Cumhuriyet Cad, 15400 Gölhisar, Burdur Turkey; 3 Alaaddin Keykubat University, Kestel Mahallesi, Konya Çimento Caddesi No: 80, Alanya, 07450 Antalya, Turkey; 4 Silopi State Hospital, Yenişehir Mah, İpek Yolu Üzeri, 73400 Silopi, Şırnak Turkey; 5 Kilis State Hospital, Kazım Karabekir Mahallesi, Abdullah Gül Bulv.Çevre Yolu Üzeri: 2/1, 79000 Kilis, Turkey; 6 Medipol University, Göztepe Mahallesi, Metin Sk. No: 4, Bağcılar, 34214 Istanbul, Turkey

**Keywords:** Distal radius, Ultrasonic evaluation, Volar plating, Dorsal cortex penetration, Screw penetration

## Abstract

**Background:**

Complications related to extensor tendons have begun to increase with the use of volar plates in the treatment of distal radius fractures. In this study, we aimed to compare four-plane radiography and ultrasonography in the evaluation of dorsal cortex screw penetration following volar plate fixation.

**Methods:**

We recruited 47 patients (33 males, 14 females, mean age 37.4 years; range 18–58 years). To evaluate dorsal screw penetration in all patients, we performed radiographs at 45° pronation, 45° supination and obtained dorsal tangential graphs at maximum palmar flexion, and a wrist lateral radiograph. Wrist ultrasonography was performed in all patients.

**Results:**

Dorsal screw penetration was detected in 12 of the 47 patients undergoing VLP application. While there was > 2 mm screw penetration in seven patients, there was < 2 mm screw penetration in five patients. On four-plane radiographs, screw penetration > 2 mm was detected in seven patients and screw penetration < 2 mm was detected in two patients. On four-plane radiography, dorsal screw penetration was not detected in three out of five patients, who were shown to have < 2 mm screw penetration by ultrasonography. In addition to perioperative four-plane radiographs are also required to detect dorsal cortex penetration in patients undergoing VLP due to distal radius fracture. However, the detection of screw penetrations < 2 mm is more likely with ultrasonography compared to four-plane radiography.

**Conclusion:**

We recommend that dorsal cortex screw penetration should be evaluated with perioperative ultrasonography.

**Trial registration:**

Research Registry, researchregistry3344, Registered 10 January 2017

## Background

Over recent years, volar locking plate (VLP) fixation in distal radius fractures has become a commonly used surgical treatment method. In VLP application, the plate is placed to the volar concave face of the radius under the pronator quadratus muscle and fixed with screws placed in a volar to dorsal direction [1]. The frequency of VLP-related complications has increased as the use of this technique has increased [2]. Since soft tissue is limited at the dorsal side, extensor tendon problems are seen more frequently, especially due to irritation caused by the tips of the screws penetrating the dorsal cortex [3, 4]. For this purpose, using screws a few millimetres shorter than the size obtained during screw size measurement is highly recommended [5]. In order to detect dorsal cortex screw penetration, 45° pronation and 45° supination radiographs are used, in addition to standard wrist lateral radiography [6]. More recently, dorsal tangential radiography of the wrist is becoming more popular to detect dorsal cortex screw penetration [7]. Besides radiography, ultrasonography (USG), another imaging technique, is also used actively. Since ultrasound signals cannot pass cortical bone, any structure or metal within the bone cannot be seen on USG. On the other hand, if a screw passes cortical bone, it can be detected very easily with USG since metal is very echoic [7, 8].

In the present study, we compared the effectiveness of four-plane radiography and USG in detecting dorsal cortex screw penetration in patients who were treated with volar plate fixation for distal radius facture.

## Methods

**Fig. 1:**
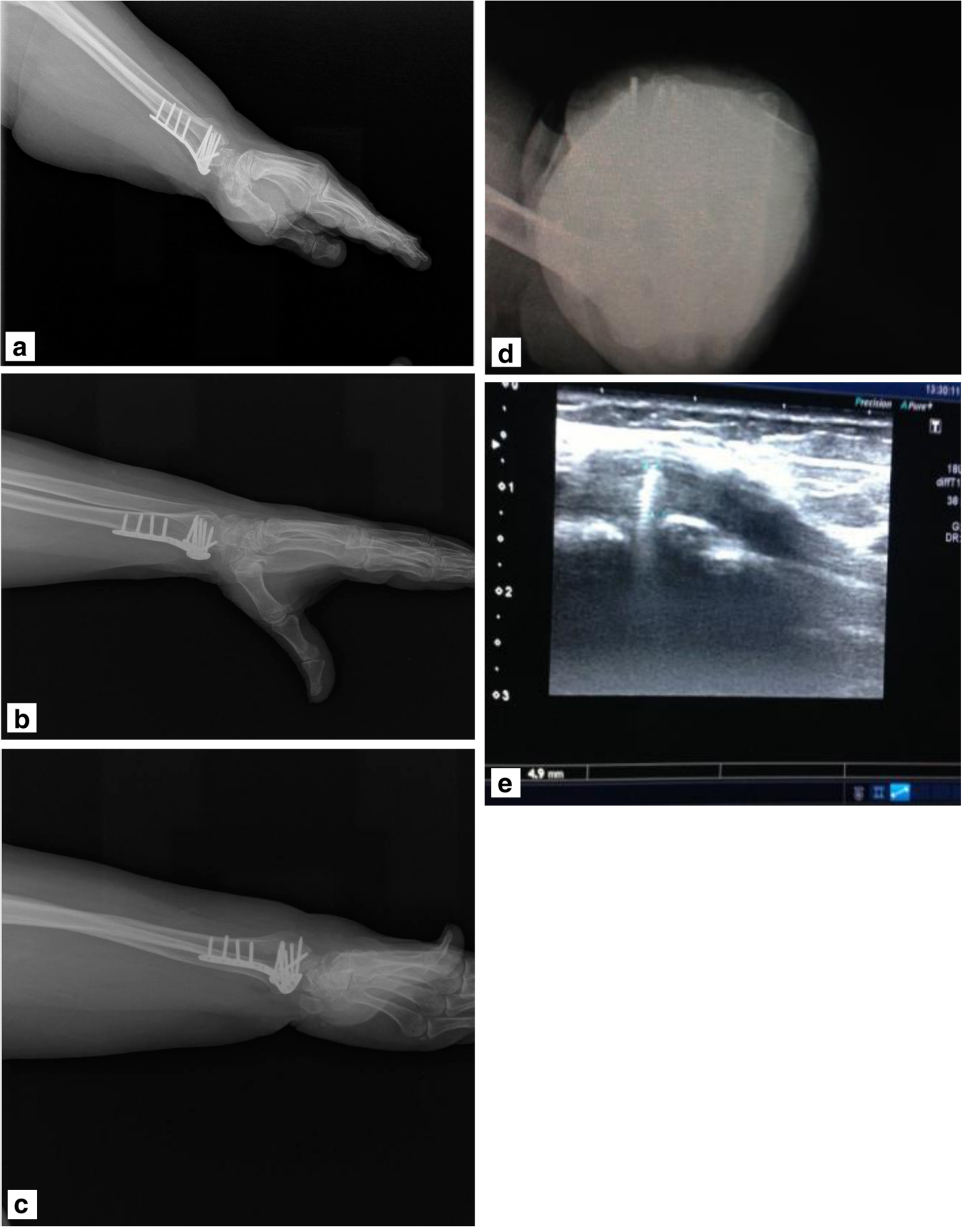
Patient number 8. Left wrist fracture, second compartment, 4.9 mm penetration. Postoperative lateral, 45° supination, 45° pronation, dorsal tangential and USG images. **a** Lateral. **b** Penetrating screw was not detectable on the 45° pronation radiograph. **c** Penetration was barely seen on the 45° supination radiograph. **d** Screw penetration on tangential radiograph. **e** 4.9 mm penetrating screw in the second compartment was seen by ultrasonography

Forty-seven patients, who underwent fixation with VLP due to distal radius fracture, between February 2011 and January 2014 were investigated. C type fractures were taken into the study. Because in these fractures, it was predicted that the screw size adjustment would be more difficult since the dorsal cortex was not intact during the operation. To evaluate dorsal screw penetration in all patients, radiographs at 45° pronation, 45° supination and dorsal tangential graphs at maximum palmar flexion were taken, in addition to wrist lateral radiographs. Since different compartments could be evaluated effectively in each graph, four graphs were taken in all patients to identify screw protrusion. A 45° supination radiograph was taken to evaluate the second compartment of wrist extensor compartments, a dorsal tangential radiograph was taken at maximum palmar flexion to evaluate the third compartment and a 45° pronation radiograph was taken to evaluate the fourth compartment [9] (Fig. 1). Tangential radiographs were taken when the shoulder was at 90° abduction and 45° internal rotation, the elbow was at 90° flexion, and the wrist was at 70–80° palmar flexion while the X-ray anterior was in the posterior direction. Tangential radiography is not possible due to the inability of patients to perform these movements effectively in the early postoperative period, but it may be appropriate to preform it during operation under anaesthesia or when the post-operative rehabilitation is completed [6, 9].

A Toshiba Aplio 500 device was used for ultrasonographic evaluation and a radiologist who was experienced in musculoskeletal system radiology performed wrist USGs in all of our patients. In total, 6 extensor tendon compartments and 12 tendons of the wrist were evaluated in sagittal and transverse planes. The compartment and the penetration depth of the screws which penetrated the dorsal cortex were identified. In addition, the effect of penetrating screws upon the nearby tendon was detected by USG. The penetration depth and the compartment level of screws identified by radiography and USG were then compared [7, 8]. We asked to radiologists for double bling evaluation. One radiologist evaluated the X-ray and the other radiologist performed the USG to the patients. The radiologist who performed the USG did not know the X-ray evaluation results.

The Number Cruncher Statistical System (NCSS, 2007) (Kaysville, Utah, USA) was used for statistical analysis. Descriptive statistics (mean, standard deviation, median, frequency, ratio, minimum, maximum) were collated and used with the Kappa agreement test, diagnostic scan tests and ROC curve area to evaluate the agreement between USG and lateral, 45° supination, 45° pronation, dorsal tangential radiographs. Statistical significance was set at *p* < 0.05.

## Results

This study was conducted on 47 patients who underwent wrist volar plate fixation between February 2011 and January 2014.

The age of the study participants varied between 18 and 59 and the mean age was 37.40 ± 9.76 years. In total, 29.8% of our study population (*n* = 14) was female and 70.2% (*n* = 33) was male (Table 1).

**Table 1:** Demographic characteristics

		Min–Max	Mean ± Standard deviation
Age		18–58	37.40 ± 9.76
		*n*	%
Gender	Female	14	29.8
	Male	33	70.2

Surgery was performed on the right wrist in 55.3% (*n* = 26) of cases and on the left wrist in 44.7% (*n* = 21) of cases. Several types of fracture were encountered: C1 in 19.1% (*n* = 9) of cases, C2 in 31.9% (*n* = 15) of cases and C1 in 48.9% (*n* = 23) of cases. Screw penetration was detected by USG in 25.5% (*n* = 12) of cases, on lateral radiographs in 10.6% (*n* = 5) of cases, on 45° supination view radiographs in 12.8% (*n* = 6) of cases, on 45° pronation view radiographs in 4.3% (*n* = 2) of cases and on dorsal tangential view radiographs in 14.9% (*n* = 7) of cases.

The predominant complaint was tenosynovitis in 14.9% (*n* = 7) of cases. Plates were removed from all patients with complaint (Table 2).

**Table 2:** Descriptive characteristics

		*n*	Percent
Site	Right	26	55.3
	Left	21	44.7
Fracture type	C1	9	19.1
	C2	15	31.9
	C3	23	48.9
Screw penetration on USG	No	35	74.5
	Yes	12	25.5
Lateral radiograph	No	42	89.4
	Yes	5	10.6
45° supination view	No	41	87.2
	Yes	6	12.8
45° pronation view	No	45	95.7
	Yes	2	4.3
Dorsal tangential view	No	40	85.1
	Yes	7	14.9
Complaint	No	40	85.1
	Tenosynovitis	7	14.9
Plate removal	No	40	85.1
	Yes	7	14.9

Upon analysis of the association between USG and radiographic evaluations of screws penetrating the dorsal wrist, we observed that dorsal tangential radiography showed the best association (*p* < 0.01). Kappa agreement level was 67.6% and ROC curve area was 79.2%. Radiographic measurements showed the highest association with ultrasound results. The next best association was with the 45° supination view in which the Kappa agreement level was 59.8% and the ROC curve area was 75% (*p* < 0.05). The next best association was wit lateral radiograph in which Kappa agreement level was 51.5% and ROC curve area was 70.8% (p < 0.05). Finally, 45° pronation radiographic measurement was not compatible with ultrasound (*p* > 0.05) (Table 3).

**Table 3:** Diagnostic screening tests and ROC curve results according to USG screw penetration

	Diagnostic scan				Kappa agreement	ROC curve		*p*
	Sensitivity	Specificity	Positive predictive value	Negative predictive value		Area	95% Confidence interval	
Lateral radiograph	41.67	100.0	100.0	83.33	0.515	0.708	0.511–0.905	0.033*
45° supination view	50.00	100.0	100.0	85.37	0.598	0.750	0.560–0.940	0.010*
45° pronation view	16.67	100.0	100.0	77.78	0.230	0.583	0.382–0.784	0.393
Dorsal tangential view	58.33	100.0	100.0	87.50	0.676	0.792	0.611–0.972	0.003**

*p < 0.05; **p < 0.01

## Discussion

Since the anatomical structure of the dorsal cortex of the distal radius is irregular and complex compared to the volar, the detection of dorsal cortex penetration at fluoroscopy is challenging. As extensor tendon compartments have a limited space, a screw penetration, which occupies a minimal space, can cause tenosynovitis at first and consequently tendon rupture with time according to the severity of the damage incurred [3, 10, 11]. We also observed tendon problems in 7 out of 12 patients (58%) who were shown to have experienced penetration of the dorsal cortex. Detecting dorsal cortex screw penetration and shortening these screws during the operation can prevent this complication. Seven of our 47 patients had symptomatic tenosynovitis. Plates were removed from all of these patients. It was seen that the extensor tendon problems were in accordance with the literature [12]. Sügün et al. detected extensor tendon problems in 14 out of 46 patients who had been treated with VLP. Of these, 12 had tenosynovitis, 1 had EPL rupture and 1 had EPL partial rupture [10]. Arora et al. reported EPL rupture in 2 out of 141 patients and tenosynovitis in 4 patients. In both studies, continuous tendon irritation was proposed as the main cause of extensor tendon problems. These authors also stated that patients with tenosynovitis might develop tendon rupture in the long term.

The detection of dorsal cortex penetrating screws is the most important preventive measure to reduce the frequency of complications related to extensor tendons in patients undergoing VLP fixation following distal radius fracture. Radiography is used most frequently to identify these screws during the perioperative and postoperative period. However, previous reports suggested that lateral wrist radiography is not sufficient to detect these screws and in addition to these radiographs, wrist radiographs at 45° pronation, 45° supination and dorsal tangential graphs at maximum palmar flexion should also be taken [4–6, 9].

In the cadaveric study of Ozer et al., wrist radiographs in four planes (lateral, 45° pronation, 45° supination and dorsal tangential) were compared in order to identify dorsal cortex penetrating screws [9]. Furthermore, these authors also reported both the compartment of the dorsal cortex penetrating screw and radiographs that showed the screw according to its length. According to this study, the second compartment is evaluated most optimally with 45° supination radiograph, the third compartment by dorsal tangential radiograph and the fourth compartment by 45° pronation radiograph. In our present study, all patients underwent four-plane radiograph imaging; when we compared the compartments and lengths of identified screws, we concluded that the second compartment was best imaged with a 45° supination radiograph, the third compartment by dorsal tangential radiograph and the fourth compartment by 45° pronation radiograph.

In the cadaveric study of Hill, wrist radiographs in four planes, the 45° supination radiograph was reported to be the most sensitive to detect the dorsal cortex screw penetration [13]. In our study, we found that the dorsal tangential radiograph of the wrist was the most correlated with the USG examination.

**Fig. 2:**
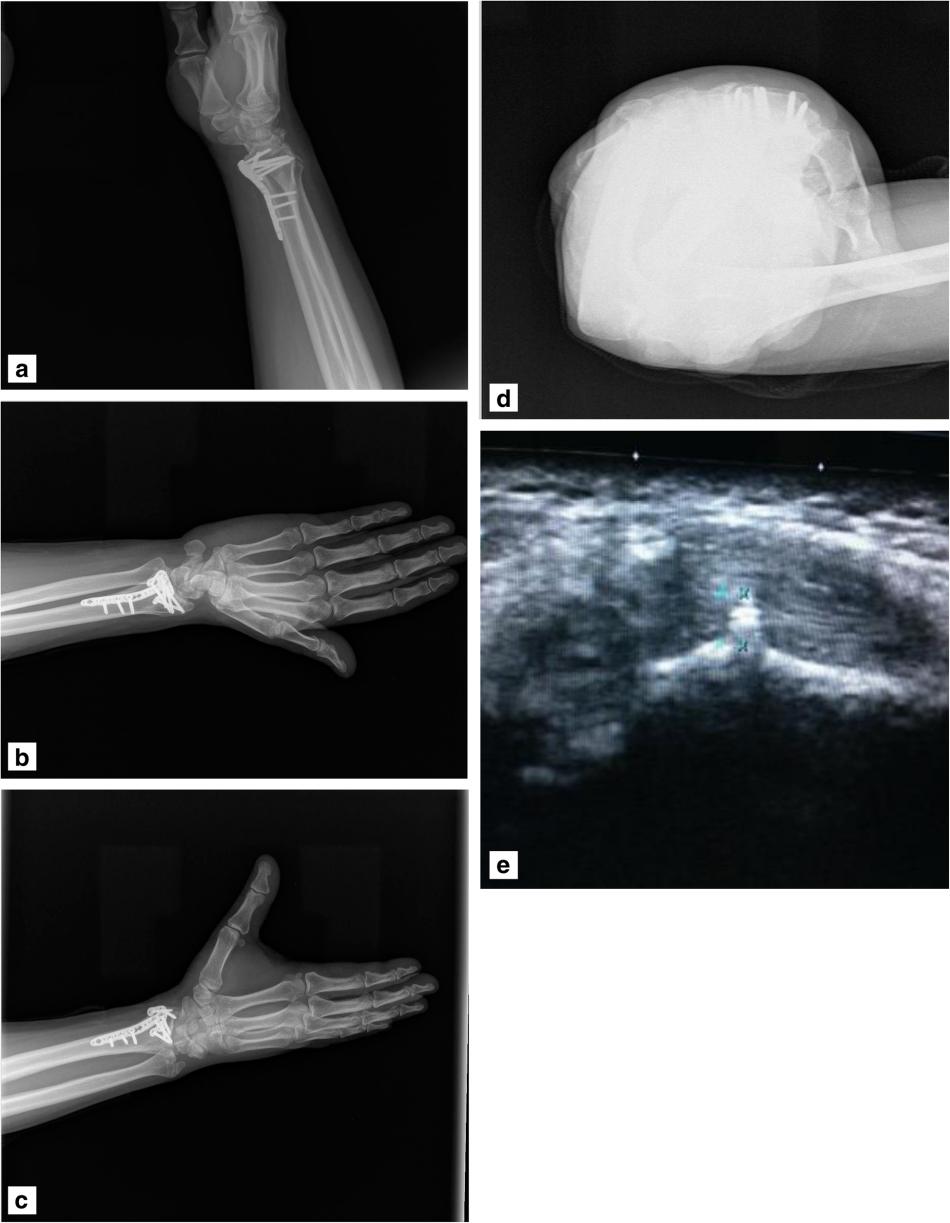
Patient number 25. Right distal radius fracture, 1.8 mm penetrating screw in the second compartment. Postoperative lateral, 45° supination, 45° pronation, dorsal tangential and USG images. **a** Lateral. **b** 45° supination view. **c** Penetrating screw was barely detectable on the 45° pronation radiograph. **d** No penetration on dorsal tangential view radiograph. **e** 1.8 mm penetrating screw in the second compartment was seen on USG

In addition to four-plane radiography, USG has also been used recently to detect soft tissue problems and screw penetration in patients treated by VLP [7, 8, 14]. Some studies have reported that USG exhibits 100% sensitivity and specificity in identifying dorsal cortex screw penetration [11]. In a cadaveric study**,** Watchmaker et al. found that dissection and USG is highly correlated in the detection of long screws penetrating the dorsal cortex; such screws are not detected by fluoroscopy after volar plate fixation. We detected dorsal cortex screw penetration in 12 out of 47 (43%) patients who had been investigated with USG. We found that USG is independent from the compartment in the detection of screw penetration. We also observed screw penetration on four-plane radiography in 9 out of 12 patients with screw penetration. The screw size and compartment in three undetected patients were 1 mm in the third compartment, 1.8 mm in the second compartment and 1.1 mm in the fourth compartment. The shortest screw size that caused symptoms was 1.8 mm in our patients (Table 4). Sügün et al. reported that screws longer than 1.5 mm cause symptoms, especially in the third and fourth compartments [12]. In our present study, we observed that USG was better than four-plane radiography, especially in detecting shorter screw penetrations (Fig. 2). Soft tissue problems in these patients were also diagnosed by USG [7]. An earlier report stated that USG is superior to MRI in the detection of soft tissue problems because of metallic artefacts in such patients.

**Table 4:** General characteristics of patients with screw penetration

Patient no	Age	Site	Radiography				USG
			Lateral radiograph	45° supination view	45° pronation view	Dorsal tangential view	
2	28	Right	–	–	–	–	1 mm, 3.comp
6	33	Left	–	+	+	+	2.1 mm, 2.comp + 1.8 mm 4.comp
8	54	Left	+	+	–	+	4.9 mm, 2.comp
11	41	Right	–	+	–	+	2.7 and 2.4 mm, 2.comp
14	38	Left	–	–	–	–	1.1 mm, 4.comp
18	44	Right	+	+	–	+	2.2 mm, 3.comp
25	46	Right	–	–	–	–	1.8 mm, 2. comp
29	42	Left	+	+	–	+	5.8 mm, 2.comp
34	34	Left	–	+	–	–	1.6 mm, 2.comp
37	36	Right	–	–	–	+	1.8 mm, 3.comp
43	48	Right	+	–	–	+	2 mm, 3.comp
45	24	Left	+	–	+	–	2.1 mm, 4.comp

Symptomatic tenosynovitis was diagnosed in seven patients with USG. As a widely available, economical easy to use, rapidly-prepared, non-invasive and dynamic diagnostic tool, ultrasound is far superior to four-plane radiography or fluoroscopy in the detection of dorsal cortex screw penetration and soft tissue problems following distal radius fractures [7, 8, 14]. Furthermore, USG is commonly available in operating rooms since anaesthetists use USG while performing regional anaesthesia.

The low number of participants can be considered as a limitation of our study. Furthermore, optimal evaluation of dorsal tangential radiographs of patients requires an adequate joint range of motion. Therefore, dorsal tangential radiographs taken in the early period may not give the desired result. These limitations should therefore be taken into consideration when interpreting our results.

Based upon this study, we propose that perioperative lateral radiography is not sufficient in the detection of dorsal cortex penetration in patients receiving VLP due to distal radius fracture. In addition to this type of radiograph, it is also necessary to take 45° supination, 45° pronation and dorsal tangential graphs. However, the likelihood of detecting dorsal cortex penetration shorter than 2 mm is higher for USG compared to four-plane radiography.

## Conclusion

Therefore, this method is been increasingly used as an effective way of detecting complications after distal radius fractures, particularly in revealing dorsal cortex screw penetration and soft tissue problems. We recommend the evaluation of dorsal cortex screw penetration with perioperative USG.

## References

[1] Orbay JL (2000). The treatment of unstable distal radius fractures with volar fixation. Hand Surg.

[2] Al-Rashid M, Theivendran K, Craigen MA (2006). Delayed ruptures of the extensor tendon secondary to the use of volar locking compression plates for distal radial fractures. J Bone Joint Surg Br.

[3] Toros T, Sugun TS, Ozsakar K (2013). Complications of distal radius locking plates injury. Int J Care Injured.

[4] Maschke SD, Evans PJ, Schub D, Drake R, Lawton JN (2007). Radiographic evaluation of dorsal screw penetration after volar fixed-angle plating of the distal radius: a cadaveric study. Hand (N Y).

[5] Smith DW, Henry MH (2004). The 45° pronated oblique view for volar fixed-angle plating, of distal radius fractures. J Hand Surg.

[6] Ozer K, Toker S (2011). Dorsal tangential view of the wrist to detect screw penetration to the dorsal cortex of the distal radius after volar fixed-angle plating. Hand (N Y).

[7] Bianchi S, van Aaken J, Glauser T, Martinoli C, Beaulieu JY, Della Santa D (2008). Screw impingement on the extensor tendons in distal radius fractures treated by volar plating: sonographic appearance. AJR Am J Roentgenol.

[8] Balfour GW (2016). Using ultrasound to prevent screw penetration. J Hand Surg Am.

[9] Ozer K, Wolf JM, Watkins B, Hak DJ (2012). Comparison of 4 fluoroscopic views for dorsal cortex screw penetration after volar plating of the distal radius. J Hand Surg.

[10] Arora R, Lutz M, Hennerbichler A, Krappinger D, Espen D, Gabl M (2007). Complications following internal fixation of unstable distal radius fracture with a palmar locking plate. J Orthop Trauma.

[11] Watchmaker JD, Daley RA, Watchmaker GP, Grindel SI (2016). Ultrasound imaging improves identification of prominent hardware in the surgical treatment of distal radius fractures: a cadaveric and prospective clinical study. J Wrist Surg.

[12] Sügün TS, Karabay N, Gürbüz Y, Özaksar K, Toros T, Kayalar M (2011). Screw prominences related to palmar locking plating of distal radius. J Hand Surg Eur.

[13] Hill BW, Shakir I, Cannada LK (2015). Dorsal screw penetration with the use of volar plating of distal radius fractures: how can you best detect?. J Orthop Trauma.

[14] Williams D, Singh J, Heidari N, Ahmad M, Noorani A, Di Mascio L (2016). Assessment of penetration of dorsal screws after fixation of the distal radius using ultrasound: cadaveric study. Ann R Coll Surg Engl.

